# Modification of Commonly Used Outcome Tools to Quantify the Patient Pain Distress Index Following Acute and Chronic Orthopedic Trauma

**DOI:** 10.7759/cureus.79930

**Published:** 2025-03-02

**Authors:** Sanjit Konda, Nathaniel P Mercer, Bradley A Lezak, Kenneth A Egol

**Affiliations:** 1 Orthopedic Surgery, Jamaica Hospital Medical Center, New York, USA; 2 Orthopedic Surgery, NYU (New York University) Langone Health, New York, USA

**Keywords:** fracture, patient-reported outcome measure, resilience, smfa, trauma, vas

## Abstract

Introduction: Patient-reported outcome measures (PROMs) are an important component of evaluating patient health and are increasingly utilized in orthopedics. However, their use remains inconsistent among orthopedic subspecialties, with only 21% of orthopedic trauma surgeons reporting regular use of PROMs in their practice. While tools for quantifying patient distress in response to pain have been developed, they are often difficult to apply due to extensive questioning and the need for prospective implementation. The purpose of this study was to propose a novel retrospective technique to measure the Pain Distress Index (PDI) using two common PROMs: the visual analog scale (VAS) and the short musculoskeletal functional assessment (SMFA).

Methods: A total of 797 patients who underwent operative repair of a tibial plateau fracture or revision of long bone nonunion were included. To quantify PDI, a linear trend line was calculated from a scatter plot of SMFA Bothersome Index (BI) vs. VAS pain scores at three months postoperatively. Reported SMFA BI was compared to predicted SMFA BI, and patients were stratified into three cohorts: “limited,” “adequate,” and “excellent” PDI.

Results: In both cohorts, SMFA Function Index scores at 6 and 12 months postoperatively differed significantly among the limited, adequate, and excellent PDI levels (p < 0.0005, p < 0.0005). Worse PDI (indicating greater distress from pain) was associated with poorer SMFA Function Index scores.

Conclusions: The combination of SMFA BI and VAS scores may serve as a useful tool to quantify PDI without requiring an additional questionnaire. “Limited” PDI was associated with poorer functional outcomes at 6 and 12 months postoperatively. This method may help predict which patients are at risk for worse functional outcomes and could serve as a retrospective proxy for resilience in future research.

## Introduction

Patient-reported outcome measures (PROMs) are an important component of evaluating patient health and are an increasingly utilized tool in orthopedics aimed at measuring outcomes and the functional status of injuries and treatments. PROMs allow physicians to quantify and understand subjective data such as function, pain levels, quality of life, and satisfaction [1], integrating patient experience into clinical decision-making [1].

Despite their broad application, the use of PROMs among orthopedic trauma surgeons remains inconsistent. Trauma patients present with a more heterogeneous spectrum of injuries compared to other subspecialties [2]. The majority of arthroplasty patients present with osteoarthritis, whereas orthopedic trauma patients have more variability in injury type and severity, making the implementation of a universal framework for collecting PROMs difficult. Furthermore, trauma patients do not present until after their injury, making the collection of baseline data more challenging [2]. As a result, fewer than 21% of orthopedic trauma surgeons report regular use of PROMs in their practice [3].

Among the various PROMs used in orthopedics, the short musculoskeletal function assessment (SMFA) is a well-validated questionnaire designed to assess physical and emotional function in patients with musculoskeletal injuries [4]. It consists of 46 questions scored on a Likert scale, with 34 questions evaluating function (Function Index, FI) and 12 questions assessing how bothered patients are by their injury (Bothersome Index, BI) [4]. The visual analog scale (VAS) for pain is another commonly used PROM, quantifying pain on a scale from 0 ("no pain") to 10 ("worst pain imaginable") [5]. While these measures provide insight into a patient's perceived pain and function, they do not inherently predict how well a patient will cope with the stresses of traumatic injury.

To address this gap, we introduce the Pain Distress Index (PDI) as a novel method for quantifying pain tolerance using commonly collected PROMs. The PDI measures how bothered a patient is by their pain relative to expected distress levels, providing insight into patient adaptation following orthopedic trauma. While this study focuses on validating the PDI, future research may explore its utility as a potential surrogate measure for resilience.

Resilience, broadly defined as a patient's ability to adapt to adverse conditions and pain, has been studied extensively in chronic conditions such as cancer, PTSD, and mental health disorders [6-12]. However, resilience is challenging to measure prospectively in orthopedic trauma patients due to the need for extensive questioning and longitudinal follow-up [13]. Traditional tools such as the Brief Resilience Scale [14] and the Resilience Scale for Adults [15] are often impractical in acute trauma settings [16]. Given these challenges, the PDI offers a retrospective and clinically feasible alternative to assess pain tolerance, which may serve as a proxy for resilience in future studies.

This study aims to develop an easily implemented method for assessing the PDI using two widely used PROMs, the SMFA BI and the VAS pain score. By leveraging these established tools, the PDI provides a way to quantify patient pain distress without requiring additional questionnaires. The second objective is to determine whether the PDI is associated with long-term functional outcomes in patients undergoing operative fracture repair, both acutely and in cases of chronic fracture nonunion. It is hypothesized that higher pain distress, as measured by the PDI, will be associated with worse functional outcomes at later follow-up. If successful, this method may allow orthopedic surgeons to better stratify patients based on pain distress and identify individuals who may benefit from additional psychological or rehabilitative support.

## Materials and methods

Patients were retrospectively identified from an institutional review board-approved prospective registry of patients who underwent operative repair of either tibial plateau fractures (AO/OTA 41) or long bone nonunion injuries between 2004 and 2020. Baseline data points, including age, sex, body mass index (BMI), smoking status, worker’s compensation status, and injury characteristics, were recorded at the time of enrollment. Patients who underwent operative repair of a tibial plateau fracture or revision surgery for long bone nonunion were eligible for inclusion. Patients were excluded if they were treated nonoperatively or did not have full follow-up at all time points. A total of 417 patients with tibial plateau fractures and 379 patients with long bone nonunion were identified as a representative sample of orthopedic trauma patients. Patients were followed at 3-, 6-, and 12-month intervals. At all follow-up visits, they completed the VAS to evaluate pain scores and the SMFA [4] to assess functional outcomes. These questionnaires were chosen for their applicability across a wide range of orthopedic injuries.

**Figure 1:**
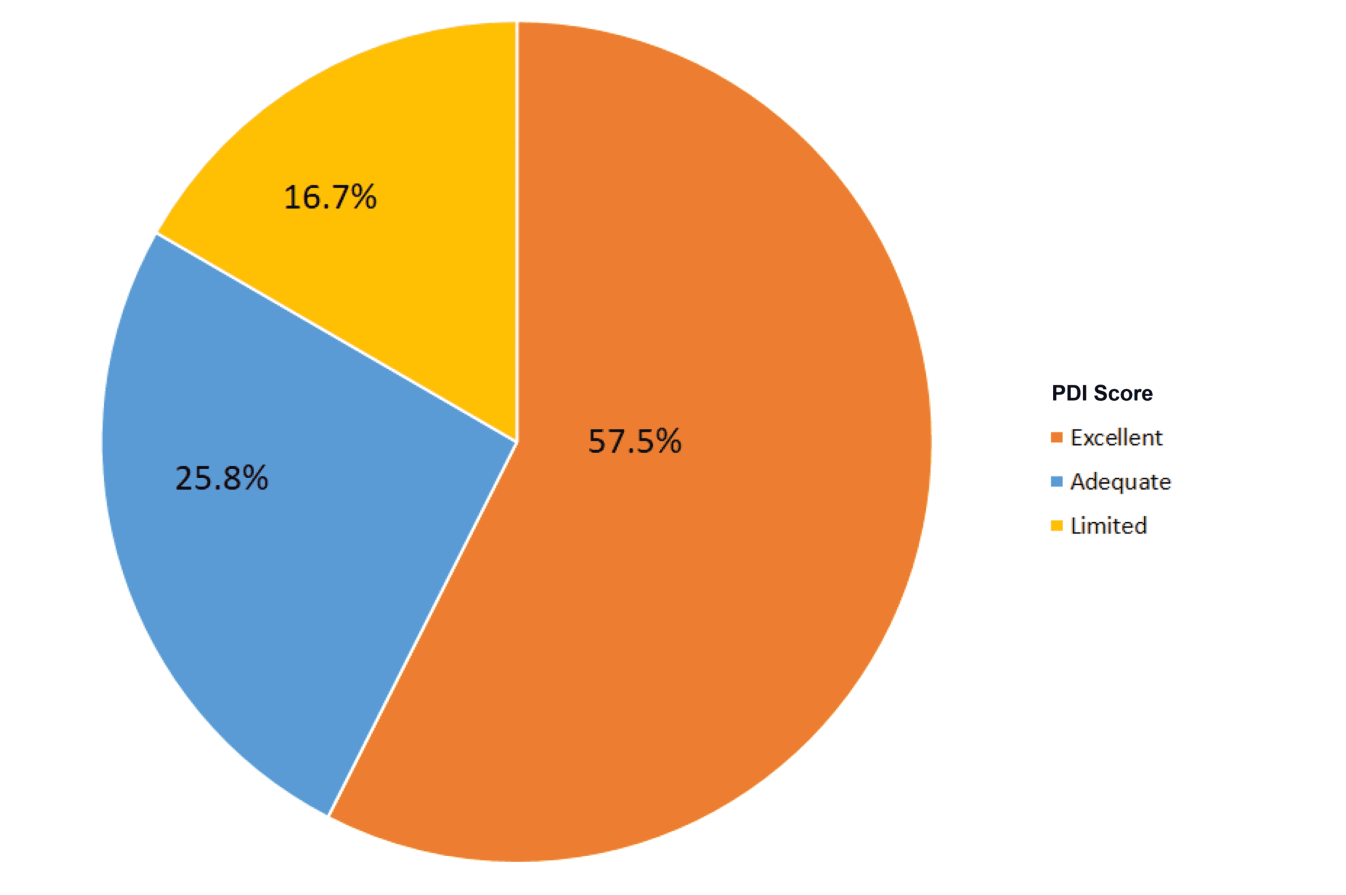
Pain Distress Index (PDI) in Nonunion Patients Distribution of poor, adequate, and excellent PDI among the nonunion cohort.

**Figure 2:**
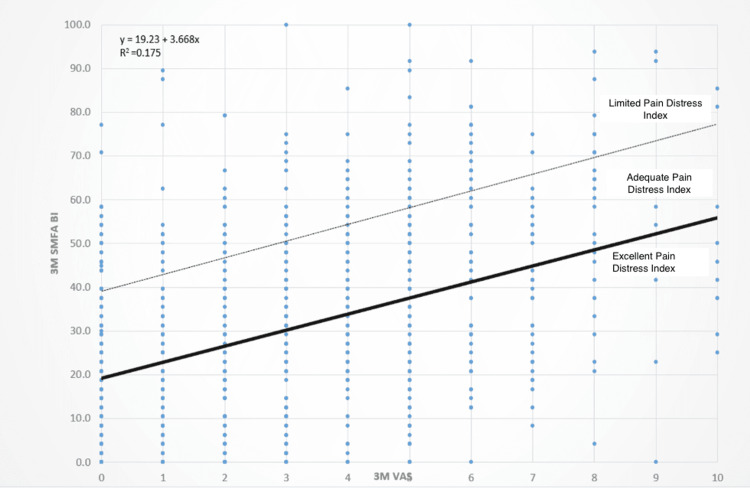
Pain Distress Index as a Function of VAS and SMFA Bother Index Scatter plot with trend lines demonstrating cutoffs for limited, adequate, and excellent Pain Distress Index based on self-reported VAS pain scores and SMFA BI at three months postoperative. VAS, visual analog scale; SMFA BI, Short Musculoskeletal Function Assessment Bothersome Index; 3M, three months.

**Figure 3:**
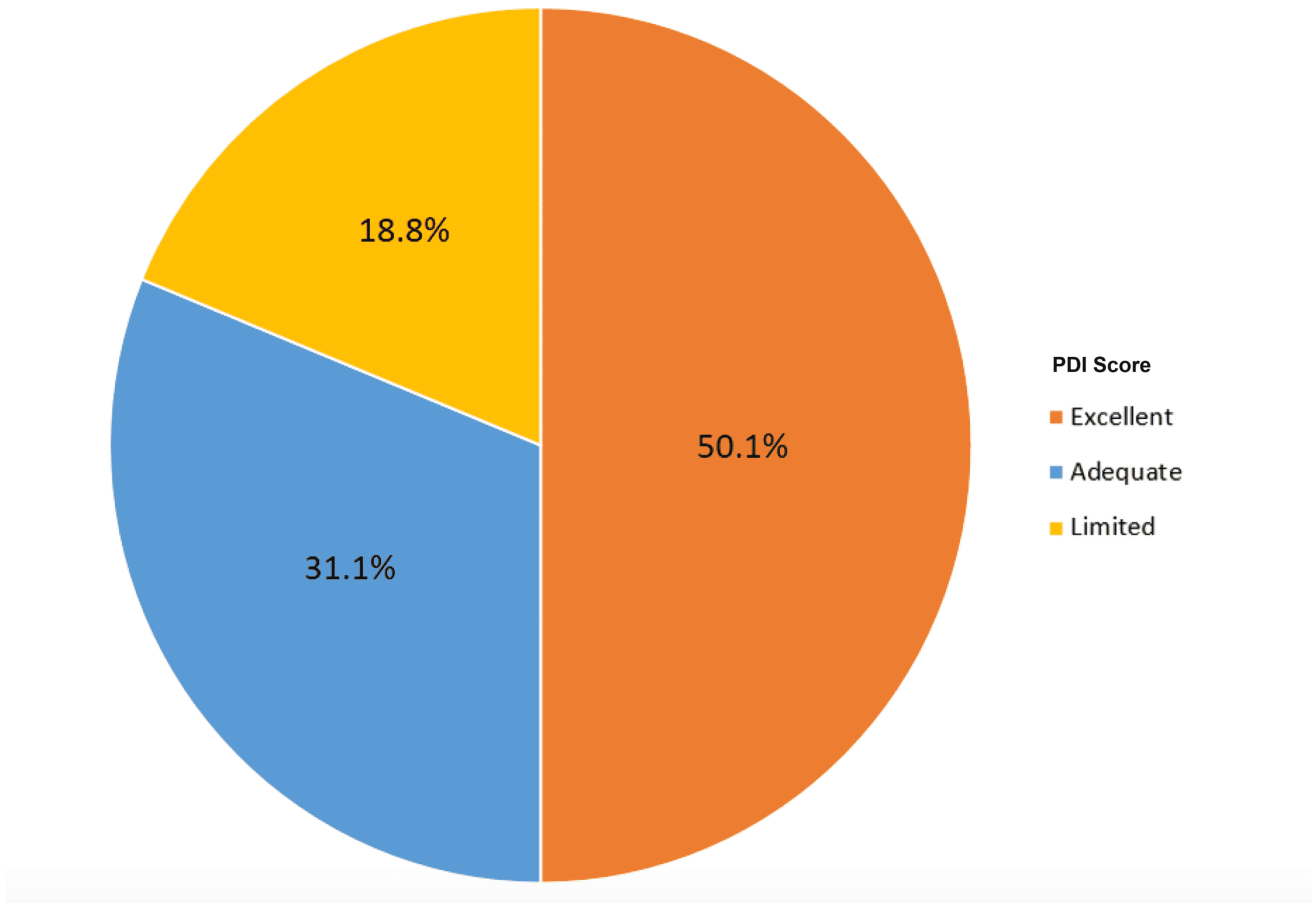
Pain Distress Index (PDI) in Tibial Plateau Patients Distribution of poor, adequate, and excellent PDI among the tibial plateau fracture cohort.

To quantify the PDI, a linear trend line was calculated from a scatter plot of the SMFA BI against the VAS pain score measured at three months postoperatively (Figure 1). This scatter plot effectively characterizes how bothered a patient is by the pain they are experiencing. Using this trend line and each patient’s true BI, patients were stratified into three groups: limited, adequate, or excellent PDI. Limited PDI was defined as having a true BI more than 20 points above the level expected based on their pain (indicating they were very bothered by their pain). Patients with a true BI between 0 and 20 points were assigned to the adequate PDI category (moderately bothered by their pain), and patients whose true BI was lower than expected were classified as having excellent PDI (not very bothered by their pain) (Figures 2, 3).

Univariate and multivariate analyses were conducted to evaluate the effect of the PDI on functional outcomes. Outcomes were assessed using the SMFA Function Index, which does not overlap with the SMFA Bothersome Index, through an analysis of variance. Baseline SMFA scores were also evaluated to determine whether PDI status was evident at baseline. Data analysis was performed using IBM SPSS Statistics for Windows, Version 25 (Released 2017; IBM Corp., Armonk, New York).

## Results

A total of 796 patients were included in this study: 417 with operatively treated tibial plateau fractures and 379 who underwent revision surgery for long bone nonunion. The average age of the entire cohort was 49.41 years, and the average BMI was 27.4. Females accounted for 49.5% of the cohort. A total of 14.1% of patients sustained an open fracture, 19.3% reported current active smoking status, and 7.8% were being treated under worker’s compensation.

Based on the linear trend line, patients were stratified into PDI categories "limited," "adequate," and "excellent" as described above. Among 417 tibial plateau patients, 78 (18.8%) had limited PDI, 130 (31.1%) had adequate PDI, and 209 (50.1%) had excellent PDI. Among 379 nonunion patients, 63 (16.7%) had limited PDI, 98 (25.8%) had adequate PDI, and 218 (57.5%) had excellent PDI.

There were no significant differences between PDI groups with regard to sex (p = 0.921), Charlson Comorbidity Index (CCI) (p = 0.168), or worker’s compensation status (p = 0.266). BMI trended toward significance (p = 0.074). Patients in the excellent PDI group were an average of 3.3 and 3.7 years younger than those in the adequate and limited groups, respectively (p = 0.021). The average age of the limited, adequate, and excellent groups was 51.5, 50.1, and 47.8 years, respectively. Females made up 50.4%, 48.3%, and 49.7% of the limited, adequate, and excellent cohorts, respectively. Both groups were healthy, with an average CCI of 0.56, 0.39, and 0.40 in the limited, adequate, and excellent groups, respectively. The average BMI of the cohorts was 28.2, 27.2, and 27.1. Patients in the excellent PDI cohort were less likely to be active smokers than those in the adequate or limited groups (p = 0.02). There was no difference in worker’s compensation status (p = 0.266) (Table 1).

**Table 1:** Baseline Demographics and Injury Characteristics (Tibial Plateau and Nonunion) BMI, body mass index; CCI, Charlson Comorbidity Index

	Limited	Adequate	Excellent	p-value
Age	47.8	51.1	51.5	0.021
Sex (% female)	50.4%	48.3%	49.7	0.921
BMI	27.1	27.2	28.1	0.074
CCI	0.56	0.39	0.40	0.168
Smoking status (% yes)	23.0%	24.1%	15.5%	0.02
Workers compensation (% yes)	8.9%	9.9%	6.4%	0.266
Open vs. closed fracture	13.5%	15.2%	14.1%	0.858

In both the fracture nonunion and tibial plateau fracture cohorts, the SMFA FI at six and 12 months was significantly different among the limited, adequate, and excellent PDI groups (Table 2). In both cohorts, PDI was inversely correlated with reported function. At 12 months postoperatively, nonunion patients with limited PDI had an average SMFA FI of 37.0 ± 23.4, while patients with adequate PDI had an average of 23.6 ± 15.5, and those with excellent PDI had an average of 15.5 ± 18.4. Tibial plateau patients with limited PDI had an average SMFA FI of 30.7 ± 21.3, while those with adequate PDI had an average of 21.6 ± 18.1, and those with excellent PDI had an average of 16.5 ± 17.3. On multivariate regression analysis, lower PDI levels at three months were also associated with worse SMFA FI scores at six and 12 months postoperatively when controlling for age, sex, CCI, tobacco use, injury pattern, and worker’s compensation status (p < 0.0005).

**Table 2:** Comparison of 12-Month SMFA FI and Baseline SMFA BI Across Pain Distress Index Cohorts for Nonunion and Tibial Plateau Fractures PDI, Pain Distress Index; SMFA FI, Short Musculoskeletal Function Assessment Function Index; SMFA BI, Short Musculoskeletal Function Assessment Bothersome Index;  12M, 12 months postoperative.

Fracture Type	Pain Distress Index Level	12M SMFA FI (Mean ± SD)	p-value	Baseline SMFA BI (Mean ± SD)	p-value
Nonunion	Limited	37.0 ± 23.4	0.023	56.1 ± 28.9	<0.0005
	Adequate	23.6 ± 16.5		47.1 ± 26.9	
	Excellent	15.5 ± 18.4		34.6 ± 24.9	
Tibial plateau	Poor	30.7 ± 21.3	<0.0005	1.7 ± 7.8	0.794
	Adequate	21.6 ± 18.1		2.3 ± 6.8	
	Excellent	16.5 ± 17.3		1.8 ± 5.3	

In patients treated for nonunion, baseline SMFA BI scores were significantly different among the limited, adequate, and excellent PDI cohorts (p < 0.0005) (Table 2). Baseline SMFA BI in the excellent PDI group was 34.6 ± 24.9, compared to 47.12 ± 26.9 in the adequate cohort and 56.1 ± 28.9 in the limited cohort. Baseline SMFA BI scores were not significantly different in patients treated for tibial plateau fractures (p = 0.794) (Table 2). It should be noted that baseline tibial plateau SMFA reports pre-injury status, whereas baseline nonunion SMFA reports pre-revision surgery status, and as such, these were evaluated separately.

## Discussion

In this study examining the effects of the PDI on patient-reported outcomes, greater PDI scores, indicating lower distress relative to pain levels, were associated with better self-reported functional outcomes at 12 months. PDI status was not evident at baseline in acute fracture patients involving the tibial plateau; however, in chronic fracture patients with a nonunion, baseline bother indices were significantly different among cohorts. This may reflect the fact that patients experiencing acute trauma have limited time to develop and display psychological adaptation to their injury. 

It is important to note that our definition of PDI is not simply the absence of bothersome feelings but rather the magnitude of bothersome feelings in relation to self-reported pain levels. Theoretically, patients with higher levels of self-reported pain should be more bothered by their symptoms. 

Kwong et al. demonstrated the equivalence of contemporaneous and retrospective patient reports in questionnaires by comparing prospectively and retrospectively completed health surveys, including hip- or knee-specific questionnaires, for patients undergoing elective hip or knee replacements. Absolute agreement was stronger for the generic PROM than for the specific PROMs. Results demonstrated that recalled health status was similar to prospective reports, suggesting that retrospectively applied tools for PDI measurement may have broad applicability [17]. This is especially valuable in an orthopedic trauma setting, where all pre-injury data is retrospective in nature.

Resilience is increasingly recognized as an important psychometric factor that may predict patient satisfaction, quality of life, and recovery outcomes across various patient populations [18]. Tokish et al. found that higher resilience scores, as measured with conventional resilience surveys, correlated with greater functional outcomes and decreased self-reported pain scores in patients undergoing elective total shoulder arthroplasty [18]. Higher resilience has been associated with lower resource utilization, including decreased hospitalizations and physician visits [19].

Sciumè et al. demonstrated that more resilient hip fracture patients self-report greater functional independence and less disability after surgery [20]. Furthermore, studies have shown that adults with greater levels of self-reported resilience are more likely to engage in higher levels of physical activity and consume healthier diets [21], while those with lower resilience scores are more likely to experience depression, fatigue, and poorer sleep quality [22].

Many factors, such as age, gender, race, preoperative mental health, and frailty, have been shown to affect surgical outcomes in orthopedic patients [12,23-25]. Age, gender, and race are nonmodifiable. Certain risk factors for frailty, such as mental health status and nutritional/diabetic status, are modifiable [26]. Preoperative mental health status is believed to have a significant effect on patient-reported pain levels [25]. Psychosocial factors of resilience are believed to be malleable, although much of the literature on this topic focuses on pediatric populations rather than adults [27]. The American Psychological Association has published several tips to increase the capacity for adaptation, such as building and utilizing social connections, maintaining physical and emotional wellness, and finding purpose in one’s work [28]. However, such strategies may have limited applicability in the setting of significant stressors associated with traumatic injury. While there is a wealth of research reporting psychological factors contributing to resilience, such as catastrophizing [29], there is little research evaluating the attempted modification of these factors.

Early identification of risk factors allows for increased anticipation of poorer postoperative outcomes. Patients suffering from orthopedic trauma, in particular, are at an increased risk for depression, anxiety, and psychological distress following their injury [30]. Data have demonstrated that orthopedic trauma patients treated with total hip or total knee arthroplasty have lower resilience scores than similar patients undergoing these procedures electively [20]. Using the SMFA BI and VAS scatter plot with trend lines as a tool to measure PDI has the potential to help identify these particularly susceptible patients. Surgeons may be able to apply this tool to stratify patients at risk for poor outcomes and intervene early in their recovery process. It may be worthwhile to involve a mental health professional, such as a psychiatrist, in patients with a limited PDI score.

Limitations of our study include the retrospective nature of this PDI evaluation and the inability to compare previously validated resilience measurement tools directly. Additionally, while patients with tibial plateau and nonunion fractures represent a variety of orthopedic trauma patients, studying a population with a wider range of injuries would allow for greater generalizability of our findings.

Future research should focus on refining and validating PDI as a retrospective proxy for resilience. By analyzing our data, we can better understand how PDI correlates with traditional resilience scales and whether it may be used as a practical screening tool to identify at-risk patients. Further study and application of this novel method of measuring PDI are required to determine to what extent this tool can be implemented in orthopedic patients after traumatic injury.

## Conclusions

The SMFA BI and VAS pain score, organized into a scatter plot with trend lines, may be a useful tool to quantify PDI. A limited PDI in orthopedic trauma patients at three months postoperatively is a predictor of worse functional outcomes at six and 12 months postoperatively. Using combined results from commonly obtained PROMs appears to predict a patient’s PDI, which may serve as a retrospective proxy for resilience. This factor may help identify patients at risk for poor functional outcomes following acute or chronic orthopedic trauma, guiding future research into resilience assessment and intervention strategies.
